# Complex effects of whole body cryostimulation on hematological markers in patients with obesity

**DOI:** 10.1371/journal.pone.0249812

**Published:** 2021-04-22

**Authors:** Joanna Wyrostek, Anna Piotrowska, Olga Czerwińska-Ledwig, Roxana Zuziak, Zbigniew Szyguła, Tomasz Cisoń, Małgorzata Żychowska, Wanda Pilch

**Affiliations:** 1 Faculty of Physiotherapy, University of Physical Education in Krakow, Krakow, Poland; 2 Institute for Basics Sciences, Faculty of Physiotherapy, University of Physical Education in Krakow, Krakow, Poland; 3 Institute of Biomedical Sciences, Department of Sports Medicine and Human Nutrition, University of Physical Education in Krakow, Krakow, Poland; 4 Department of Physiotherapy, State University of Applied Science in Nowy Sącz, Nowy Sącz, Poland; 5 Department of Sport, Faculty of Physical Education, Kazimierz Wielki University in Bydgoszcz, Bydgoszcz, Poland; Prince Sattam Bin Abdulaziz University, College of Applied Medical Sciences, SAUDI ARABIA

## Abstract

**Background:**

Adaptation, including changes in blood properties, to whole-body cryostimulation may depend on many factors, including body mass.

**Aim:**

This study investigates whether hematological parameters change similarly in a group of people with obesity and a group of men with normal body weight after 10 and 20 cryostimulation treatments.

**Methods:**

In our non-randomized trial, the participants were divided into two groups based on their body fat percentage: 14 men with a high (HBF = 29.35%) and 10 with a normal percent of body fat (NBF = 11.40%) and subjected to 20 whole body cryostimulation treatments (-120°C for 2–3 minutes). Blood samples were taken before the first and after the 10^th^ and 20^th^ cryostimulation. The following parameters were determined: red blood cells (RBC), hemoglobin concentration (HGB), hematocrit (HCT), mean corpuscular volume (MCV), mean corpuscular hemoglobin (MCH), mean corpuscular hemoglobin concentration (MCHC), platelets (PLT), red blood cell distribution width (RDW-SD), mean platelet volume (MPV), white blood cells (WBC), neutrophils (NEUT), lymphocytes (LYMPH), monocytes (MONO), eosinophils (EO) and basophiles (BASO).

**Results:**

Statistically significant differences were found in red blood cells parameters such as RBC, HCT, MCV and MCHC. Time influence was noted for HCT, MCV and MCHC. Two-way ANOVA showed a significant correlation (for time and group) for 2 paramateres: RBC and MCV. For platelet parameters statistically significant differences were found for PLT (group influence) and MPV (time and group interaction). In white blood cells parameters statistically significant differences in levels of LYMPH were noted. Higher levels were observed for HBF group.

**Conclusions:**

All observed changes were within the reference range, but hematological markers changed unevenly in people who are obese and non-obese. Therefore, it appears that an amount of fat tissue could be a factor causing the differences in adaptation to low temperature. It is suggested that 20 whole body cryostimulation sessions restore the state of homeostasis disturbed after 10 sessions.

**Trial registration:**

ACTRN 12619000524190.

## Introduction

Obesity, which is now believed to be an epidemic [1], is the cause of numerous civilization diseases due to persistent chronic inflammation [2]. Physical activity can reduce body mass and inflammation, but the introduction of physical activity in the initial phase of treatment, which focuses on the causes and effects of obesity, is often impossible in the case of people with extreme obesity. Therefore, new therapeutic methods are being sought to positively reduce obesity-induced conditions, including temperature stressor: cryostimulation.

In recent years, it was shown that cryotherapy positively affects blood circulation in the tissues. It also increases the use of fatty acids and improves immunity and antioxidant level [3, 4]. However, there is still little data on the effect of cryostimulation on blood morphology parameters in the available literature, especially in the case of people with obesity. There are few reports that refer to the effect of cryogenic temperatures on the blood composition of healthy people [5, 6] or individuals affected by ankylosing spondylitis (AS) [7]. Studies devoted to cryostimulation showed a decrease in the values of red blood cells (RBC), hematocrit (HCT), hemoglobin concentration (HGB) in both healthy participants [5] and patients suffering from AS [7]. Moreover, an increase in the number of platelets (PLT) in AS patients [7], as well as an increase in mean corpuscular volume (MCH), mean corpuscular hemoglobin concentration (MCHC) and red blood cell distribution width (RDW-SD) indicators in the case of healthy people [8] were observed. The observed changes in white blood cells (WBC) after the therapy in the cryochamber are less consistent. There were no changes [5] or increase [6] in the level of WBC. Lubkowska et al. [6] also observed changes in the level of lymphocytes (LYMPH), monocytes (MONO), neutrophils (NEUT), and eosinophils (EO).

The inconsistency of the reports may be related to the diversity of the study groups, i.e. rugby players [5], healthy men [6] or people suffering from AS [7]. Pilch et al. [9] showed a change in the white blood cell profile due to the influence of heat stress occurred only in athletes in comparison with non athletes. Żychowska and colleagues also found that depending on the level of physical activity, at the molecular level, body responds differently to heat stress [10]. Our earlier results indicate that cryostimulation can influence body composition and changes in HSP gene expression after cryostimulation depend on the number of stimulations and baseline body mass [11]. In light of these reports, it becomes reasonable to assume that the body people with obesity can react differently than the body of healthy people, or people who engage in high levels of physical activity. Therefore, the aim of our study was to evaluate and compare the changes caused by 10 and 20 sessions of whole body cryostimulation people with obesity and people with normal body mass.

## Material and methods

### The study group

**Fig 1:**
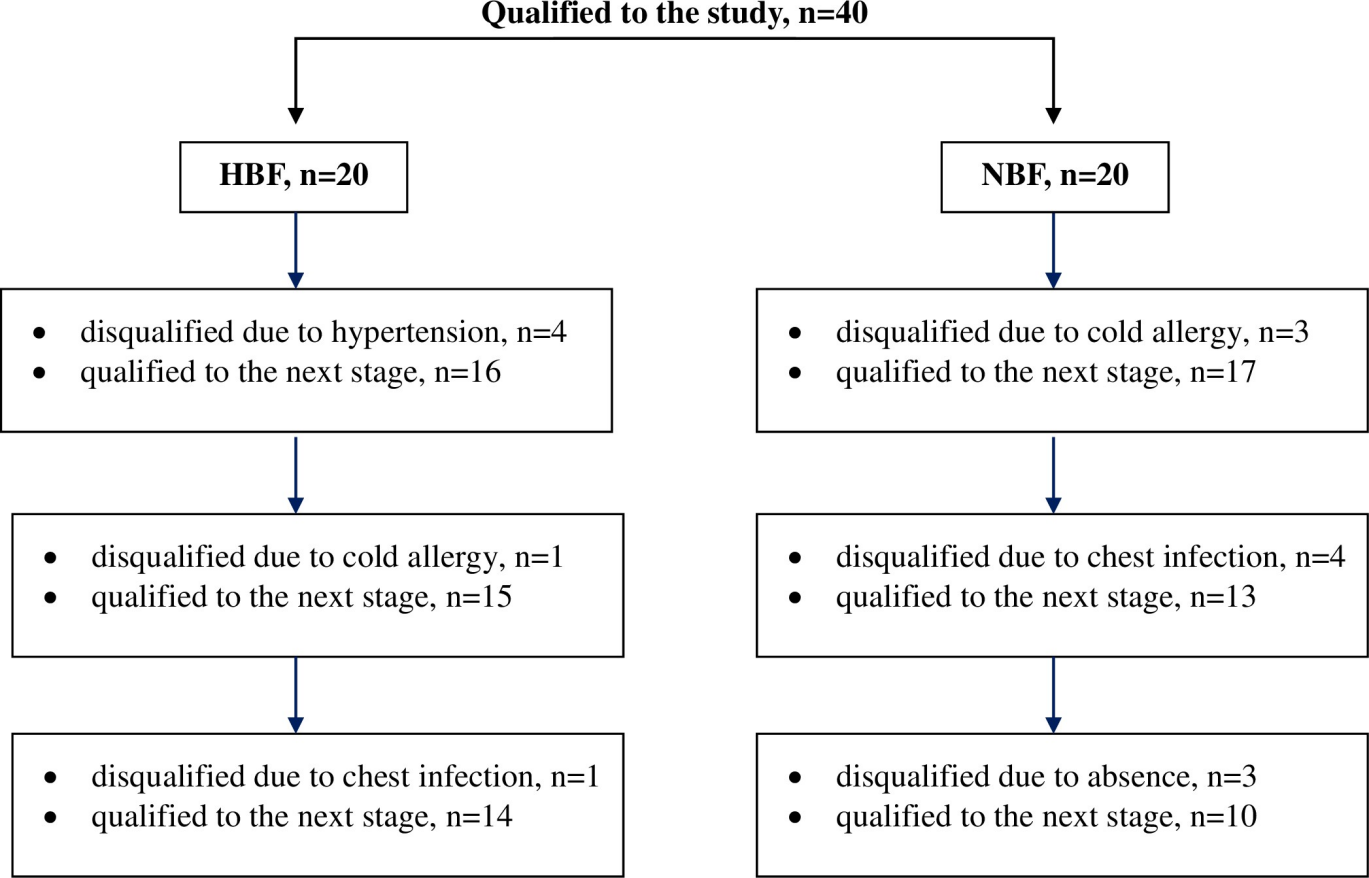
Factors disqualifying the study group during the project duration–patient flow diagram.

The study involved 40 volunteers who were interviewed and had a medical examination to exclude contraindications for cryostimulation. Recruitment for research took place from January to February 2018. The criteria for inclusion in the study included the percent body fat >26% for the high body fat (HBF) group, percent body fat 8–21% for the normal body fat (NBF) group [12], age 20–35 years old, male and no medical contraindications. The exclusion criteria included contraindications for the cryostimulation presented by Lubkowska [3] and taking anti-inflammatory drugs. Due to the presence of disqualifying factors, and adverse events (high blood pressure before entering cryochamber, respiratory tract infections, cold intolerance), only 24 young men completed the study (Fig 1). Fourteen of them were in the HBF group and 10 in the NBF group.

The study participants were informed about the aim and methods used in the study. They were also informed about the possibility to withdraw from the study at any stage of the project and they had insight into their results. All participants consented to participate in the study in accordance with the Declaration of Helsinki. The project was approved by the Bioethics Committee at the Regional Medical Chamber in Krakow (Approval number122/KBL/OIL/2017) also obtained the trial registered number (ACTRN 12619000524190). The research was submitted for registration after recruitment process, due to the lack of knowledge about the necessity of its registration. The authors confirm that all ongoing and related trials for this intervention are registered.

The characteristics of the groups subjected to further analysis are presented in Table 1.

**Table 1:** Anthropometrical characteristics of the subjects.

Parameter	HBF (n = 14)	NBF (n = 10)
	$\bar{x}$±SD	$\bar{x}$±SD
A [years]	29.64±4.13	22.10±2.33#
BH [cm]	179.34±4.72	183.41±5.27
BM [kg]	115.61±25.83	79.30±8.44#
PBF [%]	31.73±5.90	12.27±4.67#

HBF—high body fat group; NBF—normal body fat group; A–age, BH–body height, BM–body mass, PBF–percentage of body fat; #*p* ≤ 0.05 HBF vs.NBF.

Anthropometric measurements were estimated before and after the 20^th^ cryostimulation. Body mass (BM) was determined using the body composition analyzer (type IOI-353, Yawon). Percentage body fat (PBF) were determined using the electrical bioimpedance method (Bodygram PLUS, Akern BIA 101 Body Composition Analyzer). The men from the HBF group in our study were characterized by class 2 obesity with significantly increased body fat

### The procedure of systemic cryotherapy

The study participants underwent 20 whole body cryostimulation sessions (1 treatment per day, from Monday to Friday, excluding public holidays) at -120°C for 2–3 minutes in a cryochamber (Bamet KN-1) located at the Malopolska Cryotherapy Center Krakow (Poland) in months March-April 2018. The concentration of oxygen in the air of the cryochamber was kept constant at 21–22% and continuously controlled by two independent oxygen probes. In order to adapt the body to extremely low temperatures, each cryostimulation treatment was preceded by a 30-second adaptation period in the vestibule at -60°C [8]. The treatments took place under the supervision of a trained employee working in the facility. Before entering the cryochamber, the subjects had to remove glasses, contact lenses, jewelry and thoroughly dry the body to eliminate the feeling of cold. The men were dressed in shorts or sweatpants (due to the strong feeling of cold in some cases), mid-calf socks, clogs, gloves, headgear, and earmuffs. The nose and mouth were covered with a surgical mask. The study participants were instructed to move constantly and breathe slowly. The person supervising the procedure was in constant contact with the subjects. For safety reasons, each participant had their systolic and diastolic blood pressure measured before and after the treatment. The study participants were not subjected to any forms of kinesiotherapy after the session.

### Protocol deviations from study as planned

Deviations from the protocol study concerned the age of the subjects (due to the lack of recruited subjects at the age assumed in the protocol), the number of subjects, the lack of analysis of hematological indicators 7 days after the end of the experiment and the lack of biochemical analysis other indicators due to the financial limitations of the project.

### Blood samples and hematological analyses

Samples containing 6 ml of venous blood were collected from antecubital fossa veins by a laboratory diagnostician three times: before the start of the treatment in the cryochamber and within 1h after the 10^th^ and 20^th^ session with an interval of at least 6 hours after a light meal. The blood was collected using Vacumed® vacuum blood tubes and EDTA as anticoagulant. The hematologic blood parameters were determined through fluorescence flow cytometry and an analyzer Sysmex XN 9000 (Sysmex, USA) in a commercial laboratory. The tested indicators included RBC, HGB, HCT, MCV, MCH, MCHC, PLT, RDW-SD, MPV, WBC, NEUT, LYMPH, MONO, EO and BASO.

### Statistical analysis

The sample sizes needed to detect an expected effect size were calculated according to methodology indicated by Suresh et al. [13]. We used data from a pre-study in which levels of WBC were measured before and after 20 cryotherapy treatments in both groups (n = 5 per group). Sample sizes for HBF and NBF (n = 10) were determined based on the mean and SD within a group to provide 80% power and p = 0.05 (Es = 1.33; σ = 1.15).

Indices were presented as an mean (x) and standard deviation (SD) value. The type of distribution was verified using the Shapiro-Wilk test. Due to the type of distribution of values of hematological parameters, a transformation was made which allowed to convert the variables to a normal distribution and to perform repeated measures ANOVA. Statistically significant results were analyzed with use of Bonferroni post hoc test. For comparison of body composition parameters and age between groups t-student test was used. Differences were considered statistically significant for *p* value < 0.05. To assume the influence of age on the hematological parameters it was added to ANOVA as a covariate. All statistical analyses were performed with use of Statistica 13 software (StatSoft).

## Results

Table 2 shows hematological profile of the participants.

**Table 2:** Hematological profile of the participants (HBF- High Body Fat and NBF -Normal Body Fat) after the series of whole-body cryostimulation.

	Baseline		After 10^th^cryotherapy		After 20^th^ cryotherapy		ANOVA	Age
	$\bar{x}$±SD							
	HBF	NBF	HBF	NBF	HBF	NBF		
**RBC** (mln∙μl-1)	5.27 ±0.25	5.13±0.26	5.15±0.29	5.20±0.38	5.19±0.23	5.05±0.26	time*group: *p* = 0.039	*p* = 0.525
**HGB** (g∙dl-1)	15.58 ±0.99	15.64 ±1.16	15.23±0.80	15.82±1.34	15.31±0.89	15.37±1.08		*p* = 0.201
**HCT** (%)	44.84±2.51	44.74±3.04	43.37±2.04	44.41±3.68	43.89±1.87	43.74±2.86	time: *p* = 0.015	*p* = 0.323
**MCV** (fl)	85.10 ±4.36	87.19±3.26	84.26±4.06	85.34±3.06	84.64±4.29	86.56±2.83	time*group: *p* = 0.037	*p* = 0.683
							time: *p*<0.001	
**MCH** (pg)	29.58 ±1.90	30.49±1.24	29.63±2.01	30.39±1.19	29.54±1.97	30.42±1.01		*p* = 0.578
**MCHC** (g∙dl-1)	34.74±0.96	34.94±0.56	35.12±1.06	35.61±0.26	34.89±0.93	35.13±0.52	time: *p*<0.001	*p* = 0.206
**RDW-SD** (fl)	38.95 ±1.66	38.30±1.62	38.70±1.92	37.71±1.34	38.94±2.13	38.32±1.62		*p* = 0.538
**PLT** (tys∙μl-1)	281.07 ±37.33	225.00±61.21	294.14±54.84	227.00±49.73	288.93±41.53	222.20±53.26	group: *p* = 0.003	*p* = 0.291
**MPV** (fl)	10.73±0.77	11.67±1.37	10.71±0.80	11.85±1.60	10.79±0.79	11.56±1.53	time*group: *p* = 0.009	*p* = 0.179
**WBC** (tys∙μl-1)	8.09±1.37	7.04±1.09	7.96±1.40	7.74±1.46	7.78±1.55	6.93±0.68		*p* = 0.060
**NEUT** (tys∙μl-1)	4.59 ±1.17	4.33±1.42	4.64±0.99	5.02±1.67	4.31±1.25	4.04±1.01		*p* = 0.110
**LYMPH** (tys∙μl-1)	2.69 ±0.66	1.89±0.47	2.56±0.74	1.98±0.51	2.74±0.76	2.17±0.87	group: *p* = 0.021	*p* = 0.873
**MONO** (tys∙μl-1)	0.57 ±0.13	0.62±0.12	0.58±0.18	0.62±0.19	0.56±0.15	0.55±0.08		*p* = 0.443
**EO** (tys∙μl-1)	0.17±0.08	0.13±0.10	0.16±0.08	0.08±0.06	0.14±0.05	0.12±0.05		*p* = 0.094
**BASO** (tys∙μl-1)	0.04 ±0.01	0.05±0.02	0.04±0.01	0.05±0.02	0.04±0.01	0.04±0.01		*p* = 0.590

RBC- red blood cells; HGB—hemoglobin; HCT- hematocrit; MCV—mean corpuscular volume; MCH—mean corpuscular hemoglobin; MCHC—mean corpuscular hemoglobin concentration; PLT—thrombocytes, RDW—SD—red blood cell distribution width; MPV—mean platelet volume, WBC—white blood cells; NEUT -neutrophiles, LYMPH—lymphocytes; MONO—monocytes; EO -eosinophiles, BASO–basophiles.

Analysis of the results indicated statistically significant differences in red blood cells parameters such as: RBC, HCT, MCV, and MCHC (Table 2). RBC values differed in baseline vs. after 10th comparison (*p* = 0.049). The post hoc tests also confirmed significant differences in HCT values over time: baseline vs. 10th (*p* = 0.047) and baseline vs. after 20th (*p* = 0.027). For the MCV values, time was the differentiating factor (baseline vs. after 10th *p*<0.001; baseline vs. 20th *p* = 0.031 and after 10 and after 20th *p*<0.001). The group and time interaction was also shown. In HBF group MCV baseline level was significantly different from level after 10th treatment (*p* = 0.041). While for NBF we observed the following statistically significant differences: baseline vs. after 10th (*p*<0.001) and after the 10th vs. after 20th (*p* = 0.005). The MCHC level changed significantly over time: baseline vs 10th (*p*<0.001) and after 10th vs. after 20th (*p* = 0.008).

Significant differences between the study groups were confirmed (*p* = 0.003) for PLT counts. However, there was no indication of the effect of time of measurement on PLT values. For MPV, the group and time interaction is indicated. In the group of subjects with normal adipose tissue content, differences were indicated for: baseline vs. after 20th (*p* = 0.018).

**Fig 2:**
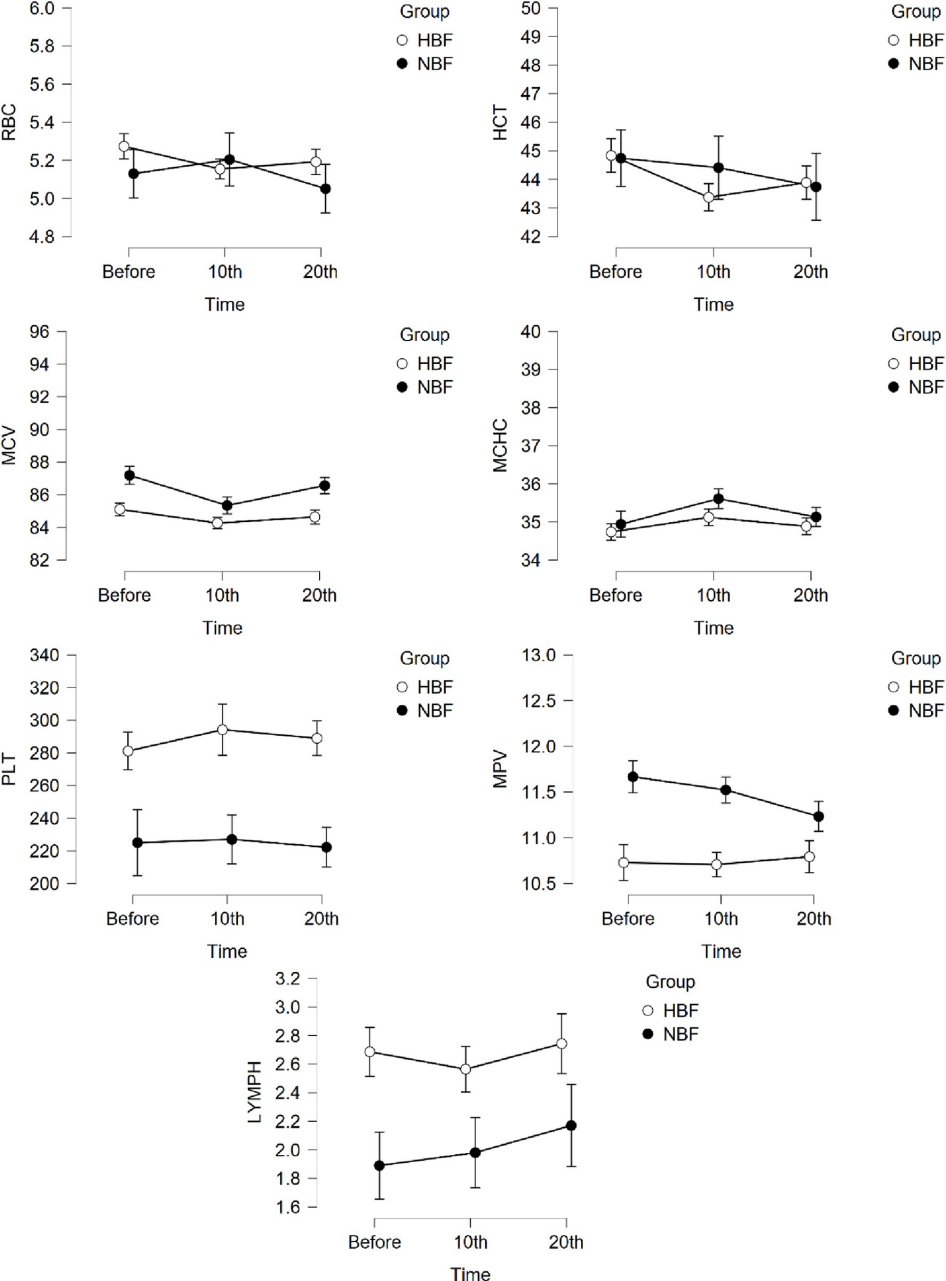
Changes in mean values of hematological parameters for which statistical significance was demonstrated.

Among the white blood cells markers no differences in the amount of neutrophils, monocytes, eosinophils and basophils between the groups were shown at the three time points. Significant differences were found for LYMPH between both groups (*p* = 0.021) with higher counts for the HBF group (Fig 2).

During the project, 16 subjects of both groups (of which 6 from the HBF group and 10 from NBF) were disqualified. For this reason, the mean age in both groups differed statistically significantly. However, the introduction of age as a covariate to the ANOVA did not show any influence of this variable on the results obtained for studied hematological parameters (Table 2).

## Discussion

Only after 10 treatments, we noticed a significant reduction (in terms of the reference range) in RBC, MCV, HCT and an increase in MCHC. In the case of men from the HBF group, significant changes were observed for RBC and MCV. The obtained results were consistent with the studies of other authors involving overweight/people with obesity [8] as well as athletes [5] and contrary to the results from studies focusing on high-ranked tennis players [14] or rugby players [5] who underwent cryotherapy. In the case of the tennis and rugby players, researchers noticed a decrease in MCHC and MCH. Ziemann and co-authors [14] showed that 5 sessions of cryotherapy were too weak a stimulus to cause any changes in the levels of RBC, HCT and MCV.

Ziemann et al. [15] demonstrated that changes in hematological parameters or lack there of after 10 treatments were associated with cardiorespiratory efficiency present in people who are obese. On the other hand, hematological changes could depend on the number of sessions in cryochamber.

Szyguła and colleagues [16] carried out an interesting study in which they compared the impact of 10, 20 and 30 sessions in the cryochamber on the changes in hematological indicators among the students of the Polish Military Academy. There was a temporary decrease in HGB, RBC and HCT levels after the 10^th^ session, no changes after another 20 sessions, and an increase compared to the baseline values after the 30^th^ cryostimulation session. Straburzyńska-Lupa and coauthors [17] obtained similar results in their study of high-ranking hockey players who underwent 18 cryostimulation treatments.

Our research showed that repeated exposure to extremely low temperatures affected MCV but did not affect RDW-SD. We observed statistically significant decrease in HBF group after 10^th^ session and in NBF group after 10^th^ (a decrease) and 20^th^ session (increase). Different results were obtained by Lombardi et al. [5] who noticed a significant increases in the MPV and RDW-SD values as a result of 14 cryostimulation treatments.

In some studies it is claimed that cryogenic therapy can have a positive effect on the immune system [6, 18], whereas others deny these claims [18]. There is little scientific evidence explaining the impact of the cryosessions in the cryochamber on the immune system in obese. As far as our study is concerned, neither 10 nor 20 systemic cryostimulation sessions caused statistically significant changes in white blood cell parameters in both groups. LYMPH counts differed significantly between groups. Higher values were observed in men from HBF group than at baseline and they remained above the values obtained in subjects from NBF after 10^th^ and 20^th^ session. In groups this parameter didn’t change significantly. Therefore, it can be concluded that the cryogenic temperature, despite differences indicated between obese and non-obese participants described above, was not a strong enough stimulus to affect the immune cells counts in people who are obese and non-obese.

In the cited study of Lubkowska et al. [8], people with obesity who performed a physical activity and whole-body cryostimulation displayed a transient decrease in the number of WBC at the end of the experiment. According to Ziemann et al. [15], no significant changes in white blood cell indicators were observed in obese who underwent 10 sessions in the cryogenic chamber. In turn, Pournot and coauthors [19] reported that there were no significant changes in the level of WBC and an increase in NEUT in runners after the 4^th^ cryostimulation treatment. Similar observations were also made by Sutkowy and colleagues [20]. The similarities concerned the level of WBC and the percentage of LYMPH and NEUT in a study involving canoeists who underwent cryotherapy sessions 2 times a day for 10 days.

Yet, Lombardi et al. [5] demonstrated no changes in WBC in the case of rugby players who were subjected to 14 sessions in the cryochamber (twice a day for 7 days). On the other hand, Lubkowska and co-authors [6] showed that 10 sessions of cryotherapy had a stimulating effect on the immune system due to a statistically significant increase in WBC in healthy men. This change was accompanied by statistically significant increases in the levels of LYMPH, NEUT, MONO, and EO. Our findings correspond with those of Lubkowska as we observed tendency in increase LYMPH level. In another study, with participation of tennis players who underwent cryostimulation sessions twice a day there was an increase in the total number of white blood cells with a simultaneous increase in the percentage of BASO observed after 5 days of treatment [14]. A similar study involving healthy and physically active men showed statistically significant changes in the level of BASO [21]. In the study by Szyguła et al. [16] cited earlier, the researchers observed a significant increase in the level of WBC after the 10^th^ and 20^th^ cryotherapy session in the cryochamber. The levels of these cells decreased after the 30^th^ cryostimulation treatment. These researchers also noticed that 20 cryostimulation sessions brought about an increase in LYMPH, while the complete series of treatments resulted in an increase in the level of MONO [16]. In our study the effect of cryogenic temperatures on the body’s immune response in either study group has not been confirmed. Furthermore, all the observed fluctuations in the white blood cell indicators were within the reference range. We noted a slight insignificant increase in LYMPH in NBF group and in HBF group cryotherapy sessions didn’t affect their counts.

Obesity accompanied by inflammation was the cause of the variation in lymphocytes number between tested groups. This allows us to assume that despite the significant age difference between study participants belonging to HBF and NBF groups, the analysis of the obtained results was possible and correctly shows the effect of cold treatment on selected hematological parameters of young men with different adipose tissue content.

During the project duration, several subjects from both groups had to be disqualified (Fig 1). The reasons for their exclusion resulted from subjects’ individual characteristics, such as cold intolerance, which couldn’t be predicted as participation in cryotherapy treatments before the start of this project was one of the exclusion criteria. Other causes were: high blood pressure and respiratory tract infections, which occurred in participants in both groups. This led to an age disproportion between HBF and NBF groups. We are not able to assume whether these causes were related to cryotherapy treatments or not. ANOVA test results showed, however, that age did not significantly affect the examined parameters (Table 2).

In our study, we clearly showed that the amount of adipose tissue has an influence on hematological markers in subjects undergoing cryostimulation. We also indicated that the number of treatments in a series is an important parameter influencing the observed changes. Our research allows for the construction of a safe treatment series for patients with morbid obesity. After 10 treatments, we observed a decrease in hematocrit and other indices that may indicate the impact of the treatments on erythropoiesis. We showed, however, that after 20 treatments, these parameters return to their previous values.

We also showed that cryochamber sessions did not significantly affect the white blood cell parameters. This should be interpreted as an evidence of non-debilitating effects on immune cells. Summarizing these observations with data on the impact of a series of treatments in the cryochamber on the pro-oxidative-antioxidant status of the plasma [22], the activity of antioxidant enzymes [22] and the expression of proteins related to iron metabolism [11], it can be indicated that these treatments are safe for patients with morbid obesity. Used in a series of at least 20 treatments, they will have a positive effect on the body composition [11], blood parameters and facilitate the patient’s preparation for the planned baritariatric procedure.

Additionally, blood morphology is often used as one of the tests performed during patients’ qualification to whole body therapy treatments. In patients with obesity, in our research and in the work by Więcek et al [23], baseline differences were indicated for white blood cell parameters in relation to healthy subjects. Our study confirmed that the treatment cycle improves these parameters in obese patients.

## Conclusions

Our results suggest that 20 cryostimulation treatments restore homeostasis disturbed after 10 treatments, since the hematological parameters were identical to the values prior to the start of the study. The demonstrated differences between groups were mostly differences that occurred in time, which may indicate a different body adaptation period to cryogenic temperatures in the case of obese and slim participants. We suggested that the amount of body fat tissue may have affected the results.

Due to the increased interest in whole-body cryostimulation, it is necessary to continue research to unambiguously address the question about the recommended number of treatments for people with obesity, whose amount of body fat and endocrine functions differ from those with a normal body weight. In this study, it has been shown that in patients with obesity, a series of 20 treatments have a more favorable effect on hematological parameters than 10 treatments. In people with a normal body weight, no such relationship has been demonstrated, so both 10 and 20 treatments are a favorable therapeutic option.

### Study limitations

The limitations of this study included a small number of subjects as well as no blood samples taken from the participants 24 hours after a cryotherapy session and 2 weeks after the last cryostimulation treatment. These blood samples would allow us to determine the duration of the effects caused by cryogenic temperatures. In addition, the study groups differed significantly in age, which was caused by disqualification factors that appeared during the study.

## Supporting information

S1 Checklist (DOCX)

S1 Data (XLS)

S1 Fig (TIF)

S1 Protocol (DOCX)

S2 Protocol (DOCX)
