# Supplementary figures and images for: Complex effects of whole body cryostimulation on hematological markers in patients with obesity
Source: PLoS One. 2021 Apr 22;16(4):e0249812. doi: 10.1371/journal.pone.0249812 (PMC8062033; doi:10.1371/journal.pone.0249812)

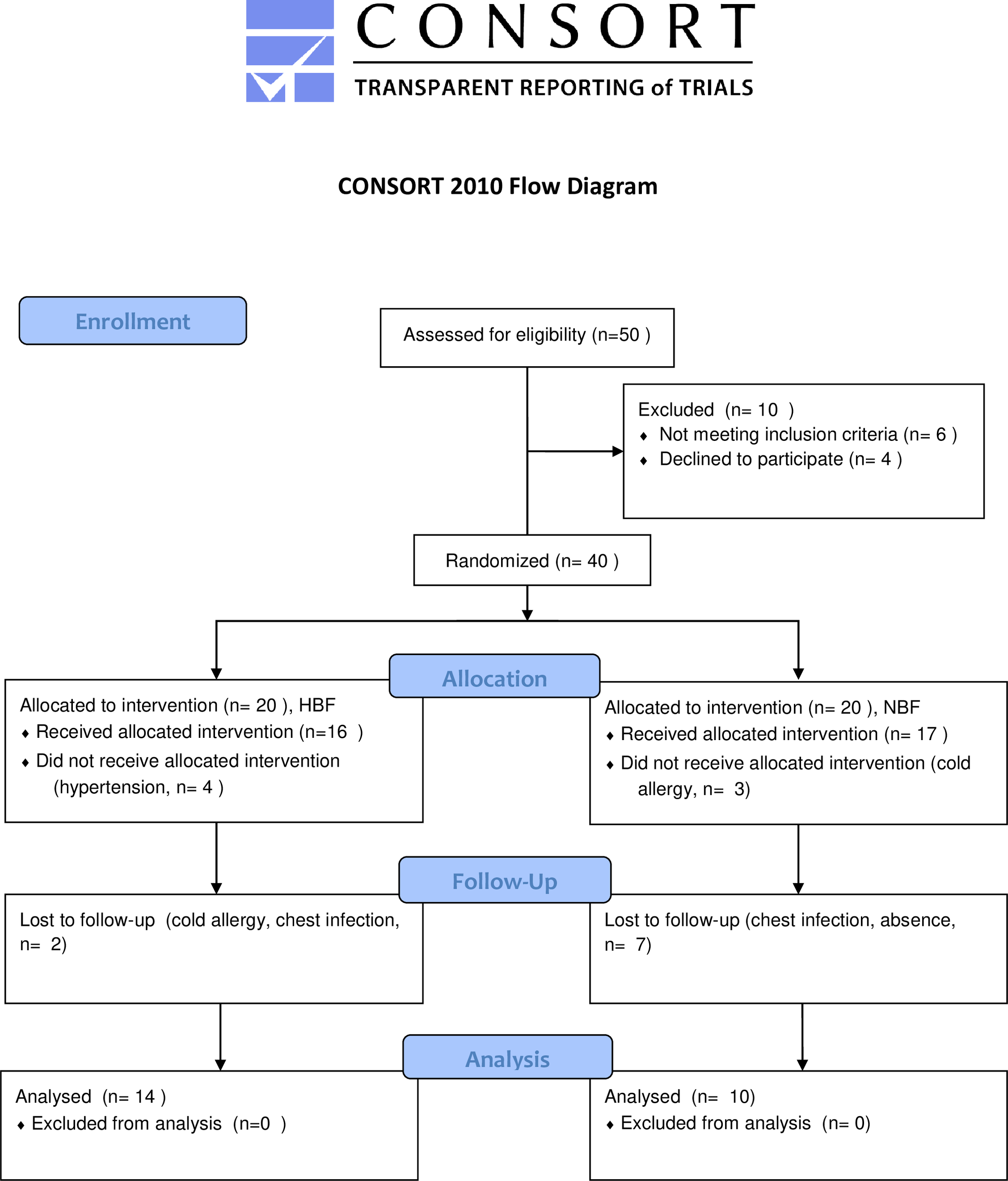

Supplement: S1 Fig — (TIF) [file pone.0249812.s003.tif]
