# Supplementary material for: Complex effects of whole body cryostimulation on hematological markers in patients with obesity
Source: PLoS One. 2021 Apr 22;16(4):e0249812. doi: 10.1371/journal.pone.0249812 (PMC8062033; doi:10.1371/journal.pone.0249812)
Supplement: S1 Protocol — (DOCX) [file pone.0249812.s004.docx]

**Study report**

**Title of project**: The influence of the whole body cryostimulation on metabolites of adipose tissue, lipid profile, level of calcidiol and morphological indicators of blood in obese men.

**Description**

Obesity is known as one of the civilization diseases and it is a serious problem of the 21^st^ century in both developing and developed countries and the percentage of people struggling with overweight shows an upward tendency. From the viewpoint of science, obesity is considered as chronic, low-grade inflammation of the body and it negatively affects its function, which often leads to more serious lesions. In recent years, studies have shown that adipose tissue apart from controlling energy homeostasis of the body also performs endocrine functions. The negative effects of obesity comprise insulin resistance, the development of diabetes mellitus type 2, cardio-vascular diseases, hypertension, atherosclerosis. In recent years, studies have shown that adipose tissue apart from controlling energy homeostasis of the body also performs endocrine functions. Adipose cells – adipocytes secrete biologically active substances - adipokines, which operate within adipose tissue, as well as further organs and tissues. In the excess adipose tissue we can observe hyperplasia of the volume of adipocytes, which causes impaired secretion of adipokines disrupted lipid homeostasis, increased susceptibility to metabolic and cardiovascular diseases and reversible changes in the cells of the immune. In addition, excess adipose tissue, reduces the bioavailability of vitamin D3.

Increasingly, cryostimulation could be used as a method of prevention and treatment of obesity. The anti-inflammatory effects of cryotherapy can alter endocrine properties of adipose tissue, because controlled hypothermia may act therapeutically by inducing physiological and biochemical changes of the body. Due to the innovative nature of the research and its specifics, available sources on the subject of whole body cryostimulation for obese people are limited and do not provide unambiguous results, because of the different number of sessions, duration of cryostimulation and the differentiation between the examined groups. So it is important to explore and continue further studies in order to use this knowledge in the struggle with obesity in adults.

In obesity, lipid profile is disturbed but cold-induced thermoregulation, increases the use of lipids, thus whole body cryostimulation has a positive effect on the lipid profile. The association between adipose tissue and vitamin D in obese is still debated by scientists. The role of vitamin D in connection with adipokines and cryogenic temperatures is still unclear, therefore these studies can provide vital new information.

The endocrine role of adipose tissue in obesity and accompanying metabolic disorders is complex and far from being fully explained, so the results obtained will allow for a broader perspective of the problem of obesity and improve preventive and therapeutic possibilities of fighting with its negative of effects. There is no doubt that the obtained results can provide valuable information for adipokines, which have not been studied yet in relation with whole body cryostimulation. The planned research will expand our knowledge of the link between selected adipokines, cytokines, lipid profile, indicators of morphological and concentration of 25(OH)D and it will help us understand the relations between specific adipocytokines, it will facilitate their use as markers of disorders associated with obesity, and thus it will enable sufficiently early prophylaxis.

**Research Project** **objective**

The aim of the proposed research will be the evaluation the effect of 20 whole body cryostimulation treatments on endocrine activity of adipose tissue, and thus the effect on the level of selected adipocytokines, reduction of inflammation in people with excessive adipose tissue and improve lipid metabolism.

**Research Methodology**

30 men aged 20-25 will be qualified to participate in the research. The control group will be consist of men with normal adipose tissue (15 people) and the experimental group will be made up of subjects with a higher percentage of body fat (> 25%). All participants will be subjected to whole body cryostimulation. Participation in the research project will be voluntary, and the absolute condition for participation will be written consent and no contraindications to systemic cryostimulation and and no medical contraindications.

Research inclusion criteria: age 20-25, males, no contraindications to whole body cryostimulation, medical qualification, normal (<23%) or increased (>25%) percentage of adipose tissue determined for age and sex.

Research exclusion criteria: cancer, advanced cardio-respiratory diseases, cardiac arrhythmias, previous blocked arteries, hypertension (>160/100 mmHg), transient ischaemic attacks, improper operation of the thyroid, diabetes, cold intolerance, frostbite, skin damage, kidney, liver or bladder disease, stroke, claustrophobia, smoking, total cholesterol (>300 mg/dL), weight-reducing diet, taking anti-inflammatory drugs.

Before commencement of the research, medical history and medical examination will be carried out in all participants, in order to exclude metabolic disorders and contraindications for whole body cryostimulation. The nutritional dietary will be interviewed and obtained data will be analyzed using the program Diet 5.0. Participants will be asked not to change their nutritional habits and not to take any physical activity during the whole experiment. Also they will be informed about the purpose of the research, methodology, possible sides effects and the possibility of cancellation of participation at every stage of the experiment.

**Research plan**

The planned project will consist of 20 sessions of systemic cryostimulation, 2 series of anthropometric tests and 4 series of biochemical tests. Measurements of anthropometric indicators will take place before first cryostimulation, while the assessment of biochemical indicators will be carried out before, during and after cryostimulation treatments.

Stages:

1. Medical examination.
2. Nutritional interview.
3. Anthropometric measurements
4. Blood collection before first cryostimulation.
5. 10 treatments whole body cryostimulation.
6. Blood collection before after 10th cryostimulation.
7. 10 treatments whole body cryostimulation.
8. Blood collection before after 20th cryostimulation.
9. Blood collection before after 20th cryostimulation.
10. Blood collection before one week after the end of the experiment.

**Whole body cryostimulation**

All participants in the experiment will be subjected to 20 sessions of whole body cryostimulation (1 treatment per day, from Monday to Friday, except on holidays) at -120^O^C for 2-3 minutes in the Malopolska Cryotherapy Centre in Krakow. Every entrance to the chamber will be preceded by a 30-second period of adaptation in the porch at -60°C. Before entering the chamber, subjects will have to remove glasses, contact lenses, jewellery and precisely dry the skin, in order to eliminate the feeling of cold during cryostimulation. A suitable outfit during cryotherapy will be: shorts, socks to mid-knee, wooden clogs, gloves and ear- and headgear. The nose and mouth will be covered with a surgical mask. At the same time, in the cryogenic chamber, 4 people will be able to stay, moving slowly, one after the other, breath slowly (short breath, long exhalation). During cryostimulation it is forbidden to talk, contact or make additional movements. Contact with the study group will be maintained by the camera and voice system. They will be instructed to leave the chamber if they feel badly or after hearing the signal that indicates the end of the treatment. Before each treatment systolic and diastolic blood pressure will be checked in order to eliminate contraindications and subjects will be informed to report every time ailments before cryostimulation.

**Anthropometric measurements**

Before first and after last cryostimulation, selected anthropometric indicators will be measured. Body weight, waist and hip circumference will be measured and the waist to hip ratio and BMI will be calculated for all subjects. In the sitting position blood pressure will be measured before each cryostimulation. For the measurement of body composition, the body composition analyzer TANITA BC 418 (93/42 EEC) will be used, operating on the principle of bioelectrical impedance.

**Blood collection**

In all participants, according to valid provisions of law, laboratory diagnostician will collect venous blood 4 times, in the morning, in fasting conditions: before commencement of whole body cryostimulation, after 10^th^ and 20^th^ sessions of whole body cryotherapy and a week after the end of the experiment.

**Marking indicators of blood**

- morphological: erythrocytes (RBC), white blood cells (WBC), lymphocytes (LYMPH), monocytes (MONO), neutrophils (NEUT), eosinophils (EOS), basophils (BASO), hemoglobin (HGB), hematocrit (HCT), mean corpuscular volume (MCV), mean corpuscular haemoglobin (MCH), mean corpuscular hemoglobin concentration (MCHC), red cell distribution width (RDW), thrombocytes (PLT), reticulocytes (RET);

- biochemical: adipokines (adiponectin, leptin, chemerin, visfatin, resistin, apelin), cytokines (IL-6, IL-1β, TNF-α, MCP-1, PAI-1, protein CRP), concentration of 25(OH)D and lipid profile (total cholesterol, fraction of LDL and HDL cholesterol, triglycerides).
