# Supplementary material for: Complex effects of whole body cryostimulation on hematological markers in patients with obesity
Source: PLoS One. 2021 Apr 22;16(4):e0249812. doi: 10.1371/journal.pone.0249812 (PMC8062033; doi:10.1371/journal.pone.0249812)
Supplement: S2 Protocol — (DOCX) [file pone.0249812.s005.docx]

**PROTOKÓŁ BADANIA**

**TYTUŁ PROJEKTU:** Wpływ ogólnoustrojowej kriostymulacji na metabolity tkanki tłuszczowej, profil lipidowy, poziom kalcydiolu oraz właściwości morfologiczne krwi u otyłych mężczyzn.

**OPIS PROJEKTU**

W obecnych czasach otyłość zaliczana jest do chorób cywilizacyjnych, a odsetek osób zmagających się z nadwagą wykazuje tendencję wzrostową. Z tego względu badania nad aktywnością tkanki tłuszczowej zyskały nowy wymiar i są istotne dla zdrowia człowieka. Z punktu widzenia nauki, otyłość uważana jest za przewlekły, niskiego stopnia stan zapalny organizmu i w perspektywie długoterminowej niekorzystnie wpływa na jego funkcjonowanie. Zazwyczaj otyłości towarzyszą liczne zaburzenia metaboliczne m.in. insulinooporność, rozwój cukrzycy typu 2, choroby układu sercowo-naczyniowego, nadciśnienie tętnicze, miażdżyca. Badania z ostatnich lat dowodzą, że tkanka tłuszczowa pełni także funkcje endokrynne. Komórki tkanki tłuszczowej - adipocyty wydzielają substancje biologicznie czynne – adipokiny, które działają w obrębie tkanki tłuszczowej, a także na dalej zlokalizowane narządy i tkanki, dlatego też wpływając na poziom adipokin, można wywierać wpływ na cały organizm. W nadmiernej tkance tłuszczowej dochodzi do rozrostu objętości adipocytów, czego konsekwencją jest m.in. zaburzona sekrecja adipokin, zakłócona homeostaza lipidów, większa podatność na choroby sercowo-naczyniowe oraz odwracalne zmiany w składzie komórek układu odpornościowego. Dodatkowo nadmiar tkanki tłuszczowej zmniejsza dostępność biologiczną witaminy D3.

Wśród metod zapobiegania i leczenia otyłości, coraz częściej w dyskusjach naukowców wspomina się o ogólnoustrojowej kriostymulacji. Jej przeciwzapalne działanie może zmieniać endokrynne właściwości tkanki tłuszczowej, gdyż kontrolowana hipotermia stanowi efektywny sposób na redukcję zapalenia poprzez indukcję zmian fizjologicznych i biochemicznych organizmu. Ze względu na nowatorski charakter badań i ich specyfikę, w literaturze znajduje się niejednoznaczne doniesienia naukowe dotyczące wpływu zabiegów w kriokomorze na poziom wybranych adipokin i cytokin ze względu na różną liczbę sesji, czas trwania zabiegu oraz zróżnicowanie między badanymi grupami. Zatem konieczne są dalsze badania, by rozwikłać liczne rozbieżności na temat kriostymulacji i otyłości. W otyłości również profil lipidowy ulega zaburzeniu, z kolei indukowana zimnem termoregulacja zwiększa wykorzystanie lipidów, dlatego ogólnoustrojowa krioterapia ma pozytywny wpływ na profil lipidowy. Związek tkanki tłuszczowej z witaminą D u otyłych wciąż jest przedmiotem dyskusji naukowców, a rola witaminy D w powiązaniu z adipokinami i wpływem temperatur kriogenicznych pozostaje wciąż niewyjaśniona, dlatego przeprowadzenie niniejszych badań, może dostarczyć nowych, ważnych informacji.

Endokrynna rola tkanki tłuszczowej w otyłości i towarzyszących jej zaburzeniach metabolicznych jest złożona i niecałkowicie wyjaśniona, dlatego otrzymane wyniki pozwolą na szersze spojrzenie na problem otyłości i poprawę możliwości prewencyjno-terapeutycznych w walce z negatywnymi jej skutkami. Nie ulega wątpliwości, iż otrzymane wyniki mogą dostarczyć cennych informacji dla adipokin, które dotychczas nie były badane w powiązaniu z zabiegami kriostymulacji. Planowane badania poszerzą wiedzę na temat powiązań między poziomem wybranych adipokin, cytokin, profilem lipidowym, wskaźnikami morfologicznymi, stężeniem 25(OH)D, a poznanie zależności miedzy określonymi adipocytokinami pozwoli na stosowanie ich jako markerów zaburzeń towarzyszących otyłości, a co za tym idzie odpowiednio wczesną profilaktykę.

**CEL BADAŃ**

Celem planowanych badań będzie ocena wpływu 20 zabiegów ogólnoustrojowej kriostymulacji na aktywność endokrynną tkanki tłuszczowej, a co za tym idzie wpływ na poziom wybranych adipocytokin, redukcja stanu zapalnego u osób z nadmierną tkanką tłuszczową oraz poprawa metabolizmu lipidów.

**MATERIAŁ I METODY BADAWCZE**

**Grupa badana**

Do udziału w badaniach zostanie zakwalifikowanych 30 mężczyzn w przedziale wiekowym wieku 20-25 lat. Grupę kontrolną będą stanowili mężczyźni z prawidłową zawartością tkanki tłuszczowej (15 osób), a grupę eksperymentalną osoby (15) z podwyższoną procentową zawartością tkanki tłuszczowej (>25%). Wszyscy uczestnicy zostaną poddani ogólnoustrojowej kriostymulacji. Udział w projekcie badawczym będzie dobrowolny, a bezwzględnym warunkiem uczestnictwa będzie pisemna zgoda oraz brak przeciwwskazań do ogólnoustrojowej kriostymulacji. Kryterium włączenia do badań to: wiek 20-25 lat, płeć męska, brak przeciwwskazań do zabiegów ogólnoustrojowej kriostymulacji, kwalifikacja lekarska, procentowa zawartość tkanki tłuszczowej (podwyższona >25% lub prawidłowa <23%). Kryterium wyłączenia z badań będą stanowiły: choroby nowotworowe, zaawansowane choroby sercowo-oddechowe, zaburzenia rytmu serca, przebyte zatory tętnicze, nadciśnienie tętnicze (>160/100 mm Hg), przemijające ataki niedokrwienne, nieprawidłowa praca tarczycy, cukrzyca, alergia na zimno, odmrożenia, uszkodzenia skóry, choroby nerek, wątroby lub pęcherza moczowego, udar, klaustrofobia, palenie papierosów, całkowita zawartość cholesterolu (>300 mg/dL), dieta redukująca masę ciała, przyjmowanie lekarstw przeciwzapalnych.

Przed przystąpieniem do badań u wszystkich mężczyzn będzie przeprowadzony wywiad lekarski i badanie lekarskie. Zostanie przeprowadzony wywiad żywieniowy, a uzyskane dane żywieniowe poddane analizie z wykorzystaniem programu Dieta 5.0. Badani zostaną poproszeni, aby nie zmieniali nawyków żywieniowych oraz nie podejmowali w czasie trwania eksperymentu żadnej aktywności fizycznej. Zostaną także poinformowani o celu badań, metodyce, możliwych efektach ubocznych i możliwości rezygnacji z uczestnictwa na każdym etapie eksperymentu.

**Plan badań**

Planowany projekt zakłada przeprowadzenie 20 sesji ogólnoustrojowej kriostymulacji, 2 serii badań antropometrycznych oraz 4 serii badań biochemicznych. Pomiary wskaźników antropometrycznych będą mieć miejsce przed rozpoczęciem zabiegów kriostymulacji, natomiast ocena wskaźników biochemicznych zostanie przeprowadzona przed, w trakcie oraz po zakończeniu zabiegów kriostymulacji.

W ramach badań przeprowadzone zostaną następujące czynności:

1. Badanie i wywiad lekarski.
2. Wywiad żywieniowy.
3. Pomiar wybranych wskaźników antropometrycznych przed rozpoczęciem kriostymulacji.
4. Pobranie krwi do badań biochemicznych przed rozpoczęciem kriostymulacji.
5. Seria 10-ciu zabiegów ogólnoustrojowej kriostymulacji.
6. Pobranie krwi do badań biochemicznych (po 10-ciu zabiegach).
7. Kolejna seria 10 zabiegów ogólnoustrojowej kriostymulacji.
8. Pobranie krwi do badań biochemicznych (po 20-stu zabiegach).
9. Czwarty pobór krwi do badań biochemicznych (tydzień od zakończenia eksperymentu).

**Metodyka ogólnoustrojowej kriostymulacji**

Uczestnicy eksperymentu zostaną poddani 20 sesjom ogólnoustrojowej kriostymulacji (1 zabieg dziennie, od poniedziałku do piątku) w temperaturze -120^O^C przez okres 2-3 minut w Małopolskim Centrum Krioterapii w Krakowie. Każde wejście do kriokomory zostanie poprzedzone 30-sekundowym okresem adaptacji w przedsionku w temperaturze -60^O^C. Przed wejściem do kriokomory badani będą musieli zdjąć okulary, soczewki kontaktowe, biżuterię, dokładnie osuszyć ciało w celu eliminacji uczucia zimna. Odpowiednim strojem podczas zabiegów kriostymulacji będą: szorty, skarpety do połowy łydek, chodaki, rękawice oraz nakrycie głowy i uszu. Nos i usta będą zakryte maską chirurgiczną. Jednocześnie w kriokomorze będą mogły przebywać 4 osoby, poruszając się wolno jeden za drugim, oddychając powoli (krótki wdech, długi wydech). Rozmowy między badanymi lub dodatkowe ruchy zostaną zabronione. Kontakt z grupą badaną będzie utrzymywany przez kamerę i system głosowy. Mężczyźni zostaną poinformowani o konieczności opuszczenia kriokomory w przypadku złego samopoczucia lub po usłyszeniu sygnału informującego o zakończeniu zabiegu. Przed każdym zabiegiem zostanie skontrolowane ciśnienie skurczowe i rozkurczowe w celu eliminacji przeciwwskazań oraz badani zostaną poinformowani, aby zgłaszać każdorazowo przed zabiegiem dolegliwości.

**Metodyka badań antropometrycznych**

Przed przystąpieniem do badań oraz po ich zakończeniu (po 20 zabiegach kriostymulacji) zostaną wykonane pomiary wybranych wskaźników antropometrycznych: masa ciała, obwód talii i bioder oraz będzie wyliczony stosunek talii do bioder i wskaźnik masy ciała (BMI). W pozycji siedzącej zostanie zmierzone ciśnienie tętnicze. Pomiar wysokości ciała zostanie wykonany wyłącznie przed rozpoczęciem kriostymulacji. Poziom wskaźników pozwalających na analizę składu ciała określony zostanie przy wykorzystaniu analizatora składu ciała TANITA BC 418 (93/42 EEC), działającego na zasadzie bioimpedancji elektrycznej.

**Metodyka oznaczeń biochemicznych**

Próbki krwi do oznaczeń biochemicznych będą pobierane 4-krotnie: przed rozpoczęciem ogólnoustrojowej kriostymulacji, po 10 i 20 sesjach kriostymulacji oraz tydzień po zakończeniu eksperymentu. Za każdym razem, rano i na czczo diagnosta laboratoryjny pobierze krew żylną. W celu określenia zmian poziomu adipokin po zabiegach ogólnoustrojowej kriostymulacji i określenia kierunku zmian u badanych zostaną oznaczone następujące wskaźniki:

- morfologiczne: erytrocyty (RBC), leukocyty (WBC), limfocyty (LYMPH), monocyty (MONO), neutrofile (NEUT), eozynofile (EOS), bazofile (BASO), hemoglobina (HGB), hematokryt (HCT), wskaźnik średniej objętości [krwinki czerwone](https://pl.wikipedia.org/wiki/Erytrocyt)j (MCV), wskaźnik średniej masy [hemoglobiny](https://pl.wikipedia.org/wiki/Hemoglobina) w [krwince czerwonej](https://pl.wikipedia.org/wiki/Erytrocyt) (MCH), średnie stężenie hemoglobiny w erytrocytach (MCHC), wskaźnik zróżnicowania w wielkości krwinek czerwonych (RDW), trombocyty (PLT), retikulocyty (RET);
- biochemiczne: adipokiny (adiponektyna, leptyna, chemeryna, rezystyna), cytokiny (IL-6, TNF-α, białko CRP), stężenie 25(OH)D oraz profil lipidowy (cholesterol całkowity, frakcja cholesterolu LDL i HDL, trójglicerydy).
